# The tetraspanin CD9 facilitates MERS-coronavirus entry by scaffolding host cell receptors and proteases

**DOI:** 10.1371/journal.ppat.1006546

**Published:** 2017-07-31

**Authors:** James T. Earnest, Michael P. Hantak, Kun Li, Paul B. McCray, Stanley Perlman, Tom Gallagher

**Affiliations:** 1 Department of Microbiology and Immunology, Loyola University Medical Center, Maywood, IL, United States of America; 2 Department of Pediatrics, Carver College of Medicine, University of Iowa, Iowa City, IA, United States of America; 3 Department of Microbiology, University of Iowa, Iowa City, IA, United States of America; University of Maryland School of Medicine, UNITED STATES

## Abstract

Infection by enveloped coronaviruses (CoVs) initiates with viral spike (S) proteins binding to cellular receptors, and is followed by proteolytic cleavage of receptor-bound S proteins, which prompts S protein-mediated virus-cell membrane fusion. Infection therefore requires close proximity of receptors and proteases. We considered whether tetraspanins, scaffolding proteins known to facilitate CoV infections, hold receptors and proteases together on cell membranes. Using knockout cell lines, we found that the tetraspanin CD9, but not the tetraspanin CD81, formed cell-surface complexes of dipeptidyl peptidase 4 (DPP4), the MERS-CoV receptor, and the type II transmembrane serine protease (TTSP) member TMPRSS2, a CoV-activating protease. This CD9-facilitated condensation of receptors and proteases allowed MERS-CoV pseudoviruses to enter cells rapidly and efficiently. Without CD9, MERS-CoV viruses were not activated by TTSPs, and they trafficked into endosomes to be cleaved much later and less efficiently by cathepsins. Thus, we identified DPP4:CD9:TTSP as the protein complexes necessary for early, efficient MERS-CoV entry. To evaluate the importance of these complexes in an *in vivo* CoV infection model, we used recombinant Adenovirus 5 (rAd5) vectors to express human DPP4 in mouse lungs, thereby sensitizing the animals to MERS-CoV infection. When the rAd5-hDPP4 vectors co-expressed small RNAs silencing *Cd9* or *Tmprss2*, the animals were significantly less susceptible, indicating that CD9 and TMPRSS2 facilitated robust *in vivo* MERS-CoV infection of mouse lungs. Furthermore, the S proteins of virulent mouse-adapted MERS-CoVs acquired a CD9-dependent cell entry character, suggesting that CD9 is a selective agent in the evolution of CoV virulence.

## Author summary

Enveloped viruses rank among the most dangerous zoonotically emerging pathogens. Their cell entry often requires multiple transmembrane proteins in the target cell, which may interact with each other to promote viral-cell membrane fusion. Susceptibility to virus infection may correlate with these transmembrane protein interactions. Here we report that the scaffolding tetraspanin protein CD9 links the receptor for MERS-CoV to a membrane fusion-activating protease called TMPRSS2, forming a complex that promotes rapid and efficient infection. The related human CoV strain 229E was also facilitated by CD9, indicating that multiple CoVs depend on tetraspanin-directed clustering of receptors and proteases for efficient cell entry. Reliance on CD9 specifically applied to virulent, *in vivo* mouse lung-adapted MERS-CoVs, suggesting that the most efficient virus entry pathways in natural respiratory CoV infections are facilitated by tetraspanins. This suggestion was reinforced by selectively regulating gene expression *in vivo*, using recombinant adenovirus transducing vectors. The findings demonstrated that CD9 facilitated MERS-CoV infections in mice.

## Introduction

Enveloped virus-cell entry requires glycoprotein-catalyzed fusion of viral and host cell membranes. These viral fusion glycoproteins are catalytically-inactive on virus particles and become triggered to mediate membrane mergers only in response to cellular and environmental factors. This triggering process ensures that virus-cell entry occurs at the appropriate time and place. The triggering factors include host cell receptors, endosomal acids, and proteases. Many viruses require a single, soluble trigger, for example, influenza A virus fusion proteins are triggered by protons within the target-cell endosome [1]. Other viruses require two triggering agents, for example, avian sarcoma leukosis virus fusion proteins are partially advanced into fusion-catalyzing forms after binding to host cell receptors, and then fully execute fusion after being exposed to endosomal protons [2]. Most CoVs also require two triggering agents, receptor binding and proteolytic cleavage, with the proteolysis taking place on receptor-bound viral ligands [3]. As many of the CoV-cleaving proteases are transmembrane-anchored, it follows that CoV-susceptible cells might have the two triggering agents, receptors and proteases, in close proximity. Here we considered whether the two CoV entry factors are coalesced on cell surfaces to facilitate infection, and whether particular host cell features are required to juxtapose the two entry factors.

The CoV receptors are all transmembrane glycoproteins. Their presence is a defining feature of host cell susceptibility to infection [4–7]. Proteases, the second required determinants of host susceptibility, are variable in type and subcellular location [8], with proteases in the type II transmembrane serine protease (TTSP) family figuring prominently [8–10]. TTSP family members, most notably the transmembrane protease serine type 2 (TMPRSS2), can cleave CoV fusion glycoproteins (termed spike [S] proteins), into unlocked, fusion-catalyzing forms [8, 9, 11] at the cell surface and facilitate a rapid, “early” entry. Studies examining HIV [12] and influenza [13] glycoproteins indicate that multiple adjacent fusion glycoproteins must be activated in order to successfully complete the fusion reaction. Assuming similar requirements for CoV fusion, it is likely that multiple S proteins need simultaneous receptor engagement and sufficient proteolytic cleavage to form an activated cluster that can pull opposing membranes together. Thus, fusion likely occurs in regions of the cell membrane with a relatively high local concentration of these entry factors.

Recent studies have confirmed that TTSPs are concentrated into punctate locations on the cell surface, in association with tetraspanin scaffolding proteins [14]. Tetraspanins comprise a family of proteins with four transmembrane spans and two extracellular loops [15]. Tetraspanins interact with other tetraspanins [16] and with other membrane-associated proteins [17, 18], including transmembrane proteases [19, 20], to form “webs” of interacting proteins [15]. There is evidence that these tetraspanin webs are locus points for CoV-cell entry, as tetraspanin-specific antibodies protect several cell types from CoV infection [14]. However, it remains unclear if individual tetraspanin proteins facilitate CoV entry and what function they have in determining viral entry routes. As there are demonstrations that the tetraspanin CD9 interacts with the MERS-CoV receptor dipeptidyl peptidase 4 (DPP4) [21, 22] and hints of similar CD9 interactions with the HCoV-229E receptor aminopeptidase N (APN) [23], we hypothesized that CD9 is necessary to bring these virus receptors to TTSP-enriched regions on the cell surface.

No study to date has determined the relative importance of individual tetraspanins and TTSPs to MERS-CoV infection in the lung environment. Indeed, there are 34 human tetraspanins and at least 17 members of the TTSP protease family [24] as well as several soluble extracellular proteases, such as elastases [25], that may be expressed in the lung parenchyma. While studies suggest that TMPRSS2 can trigger MERS-CoV in cell culture [9, 25], it is unclear whether CD9 or TMPRSS2 stand out *in vivo* as single proviral members of their respective protein families. Therefore, we set out to determine whether, and to what extent, MERS-CoV utilizes CD9 and TMPRSS2 during *in vivo* infection. To this end, we established a mouse model in which virus-resistant mice are rendered susceptible to MERS-CoV infection by expression of human *DPP4* (h*DPP4*). The system utilizes a recombinant adenovirus type 5 (rAd5) to transduce the h*DPP4* gene, thereby sensitizing only the Ad5-transduced lung cells to subsequent MERS-CoV infection [26]. The rAd5-h*DPP4* vectors were engineered to include additional genes encoding the potential virus-promoting factor human TMPRSS2 [9] or potential virus-restricting factors, in the form of shRNAs targeting murine *Tmprss2* and *Cd9*. We considered the rAd5-h*DPP4* system to be especially valuable, as MERS-CoV infection can only occur in cells expressing hDPP4 and, thus, only in cells simultaneously expressing the putative virus-promoting or virus-restricting factors.

Using the dual-expressing rAd5 vectors, as well as tetraspanin knock-out cell lines, we evaluated the roles for CD9 and another related tetraspanin, CD81, in dictating receptor clustering with proteases and in promoting CoV infection. Our results indicate that a CoV-cell entry portal is a multipartite complex that minimally includes the virus receptor, a virus-activating protease, and one or more tetraspanins. These complexes are responsible for the majority of MERS-CoV entry in lung cells. Furthermore, CD9 facilitated cell entry by MERS-CoV spikes that were adapted for lung virulence, but CD9 provided no support to cell culture-derived, avirulent spike-mediated cell entry. These data establish tetraspanins as factors controlling early entry events in pathogenic CoV infections.

## Results

### Production of tetraspanin knockout cell lines

**Fig 1:**
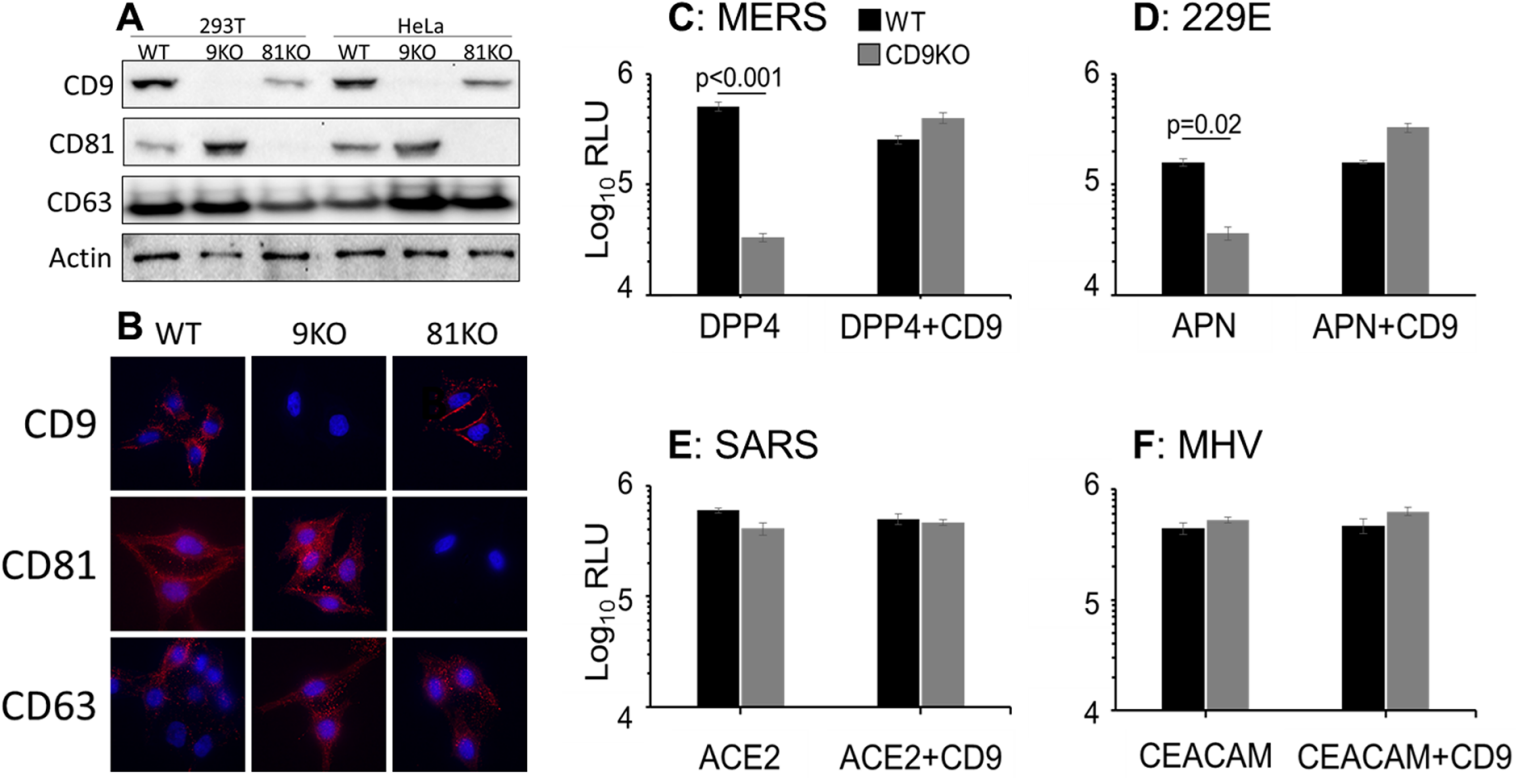
CoV-S mediated entry into tetraspanin KO cells. (A) Western blot analysis of 293T and HeLa clonal cell lines. Actin and the tetraspanin CD63 are used as loading controls. (B) Immunofluorescent analysis of HeLa clonal cell lines. Unpermeabilized cells were incubated with primary antibodies against CD9, CD81 or CD63 as indicated. 293T WT or CD9KO cells were transfected with the appropriate receptors and CD9 where indicated. These cells were transduced with viruses pseudotyped with S proteins from MERS (C), 229E (D), SARS (E), or MHV (F). Pseudovirus transduction was measured by luciferase assay.

Tetraspanins CD9 and CD81 are known to influence enveloped virus entry [14, 27, 28]. Therefore, we used CRISPR/Cas9 technology [29] to eliminate these tetraspanins from cells, with the expectation that this would affect cell susceptibility to CoVs. 293T and HeLa cells were transfected with Cas9/guide RNAs targeting *CD9* or *CD81*, selected for puromycin resistance, and cloned by endpoint dilution. All KO cell lines grew equivalent to parallel “WT” control clones, and the only observable distinctions were with the CD9KO cells, which adhered less tightly to plastic than WT or CD81KO cells. Western blot analyses of the WT and KO clones confirmed the absence of CD9 or CD81, with maintenance of a control tetraspanin CD63 (Fig 1A). Interestingly, CD81 levels were highest in CD9KO cells and CD9 levels were low in CD81KO cells, possibly due to heterotypic CD9-CD81 interactions influencing tetraspanin stability. Lower-resolution immunofluorescent assays (IFAs) of umpermeabilized cells showed similar cell-surface CD9 in WT and CD81KO cells, confirmed the absence of the respective tetraspanins in KO cells, and demonstrated that CD63 distribution remained unchanged in all cell lines (Fig 1B).

### CD9 promotes virus entry directed by MERS-CoV and 229E-CoV S proteins

To determine whether the tetraspanins CD9 or CD81 operate in CoV entry, we utilized HIV pseudoparticle (pp) transduction methodologies, which allow for a specific focus on the virus-cell entry stage. We first sensitized the cells to transduction by overexpressing CoV receptors, then transduced cells with the respective CoVpps. Relative to WT cells, CD9KO cells were 94% less susceptible to MERS (EMC strain) pp transduction (Fig 1C), and 80% less susceptible to 229Epp transduction (Fig 1D). However, CD9KO cells remained fully susceptible to SARSpp or MHVpp transduction (Fig 1E and 1F). CD9 complementation restored susceptibility to MERSpp and 229Epp transductions (Fig 1C and 1D). CD81 KO cells were fully susceptible to all four of the CoVpps (S1 Fig). These data identify an individual tetraspanin, CD9, as an entry factor for a CoV.

To determine whether receptor overexpression might have contributed to CD9 dependence, MERSpps were also transduced into cells containing endogenous CoV receptor levels. Consistently, CD9 was necessary to fully sensitize cells to MERSpps (S2 Fig), indicating that CD9 proviral activity was independent of hDPP4 receptor levels. However, CD9 was not necessary for MERSpp transduction into cells overexpressing TMPRSS2, a MERS-CoV activating protease (S2 Fig). The fact that TMPRSS2 obviated the CD9 requirement indicated a role for CD9 in proteolytic activation of CoV entry.

### CD9 directs selected CoV receptors into tetraspanin-associated membrane microdomains

The observation that a single tetraspanin family member, CD9, promoted cell entry for some, but not all CoVs, suggested that CD9 interacts with one or more MERS-CoV and 229E-CoV entry factors. We considered whether CD9 associates with DPP4 and APN, the MERS-CoV and 229E-CoV receptors, or with TMPRSS2. Furthermore, we considered whether CD9 does not interact with ACE2 and CEACAM, the receptors for CD9-independent SARS and MHV-CoVs. This was first investigated through biochemical isolation of tetraspanin-enriched membrane fractions, and detection of tetraspanin-associated receptors and proteases. To this end, CD9 or CD81 KO cells overexpressing CoV receptors or TMPRSS2 were surface-biotinylated, and tetraspanins were liberated from cells using zwitterionic CHAPS detergent, which solubilizes cell membranes while leaving tetraspanin-mediated protein interactions largely intact [30]. Low-Density (LD) fractions, with ρ<1.13 g/ml, were then separated from High-Density (HD) CHAPS-solubilized proteins on sucrose density gradients [31]. As evaluated by streptavidin pull-down and western immunoblotting, the LD sucrose gradient fractions from CHAPS-solubilized cells contained nearly 100% of cell-surface tetraspanins (S3 Fig), but only ~ 20% of the surface-biotinylated plasma membrane proteins [14], indicating efficient tetraspanin segregation into LD fractions.

**Fig 2:**
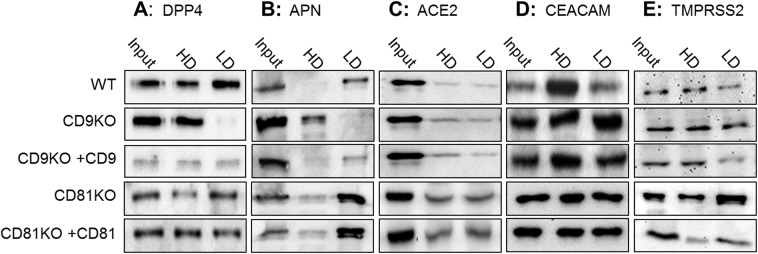
Association of CoV entry factors with CHAPS-resistant membranes in the presence or absence of CD9 or CD81. 293T WT, CD9KO, or CD81KO cells were transfected with the CoV receptors DPP4 (A), APN (B), ACE2 (C), CEACAM (D), or the protease TMPRSS2 (E). KO cells were also complemented with the appropriate tetraspanin. Cell-surface proteins were biotinylated before cells were lysed in cold CHAPS and cleared lysates were subjected to ultracentrifugation. Cell surface proteins were isolated by streptavidin pulldown and analyzed in high density (HD) and low density (LD) fractions by western blot.

Strikingly, the LD fractions from WT control cells contained ~60% of cell-surface DPP4, while LD fractions from CD9 KO cells completely lacked DPP4 (Fig 2A, rows 1 and 2). Complementing CD9 back into CD9KO cells restored LD-associated DPP4 (Fig 2A, row 3). The presence or absence of CD81 had no effect on DPP4 distribution between HD and LD fractions (Fig 2A, rows 4 and 5). Similar results were observed with the 229E receptor APN (Fig 2B). By contrast, CD9 and CD81 expression had little effect on the distribution of ACE2, CEACAM, or TMPRSS2, all of which distributed about equally between LD and HD fractions (Fig 2C–2E). These data indicated that DPP4 and APN positioning into tetraspanin-enriched membranes required CD9. The fact that CD9 repositioned DPP4 and APN, but not ACE2 or CEACAM, correlated with the fact that CD9 promoted the entry of DPP4- and APN-utilizing MERS and 229E viruses, but not ACE2- or CEACAM-utilizing SARS and MHV viruses (Fig 1).

### CD9 facilitates close proximity of DPP4 and TMPRSS2 at the cell surface

The evaluations of membrane fractions suggested that CD9 might localize the MERS-CoV and 229E-CoV receptors close to virus-activating TMPRSS2. To determine whether CD9 facilitates specific interactions between DPP4 and TMPRSS2, we analyzed intact tetraspanin microdomains *in situ*. We performed proximity ligation assays (PLAs), which can determine whether two or more transmembrane proteins are adjacent [32]. In PLAs, antibodies differentially tagged with oligonucleotide probes are applied to cells, and their close spacing (<40 nm) allows for probe hybridization into DNA polymerization templates, which provide a locus point for fluorescent DNA synthesis [33]. PLAs have been used to identify interactions between tetraspanins and their partner proteins [34, 35] and we used this method to analyze clustering of two tetraspanin partner proteins.

HeLa cells were chosen for PLAs because their relatively flat morphology facilitated quantification of fluorescent foci, and because our quantitative reverse transcriptase–PCR measurements revealed endogenous expression of CD9, DPP4 and TMPRSS2 (S1 Table). Notably, CD9 transcripts were plentiful in the HeLa cells (~10 times more abundant than the reporter gene HPRT), while DPP4 and TMPRSS2 were scarce (~50 times less abundant than HPRT, and 5- to 100 times less than that found in several human airway epithelia-derived cell cultures (see S1 Table)). Thus, we presumed that, with HeLa cells, we could readily detect a CD9-directed coalescence of sparse DPP4 and TMPRSS2.

We performed PLAs on unpermeabilized CD9KO HeLa cells, using primary antibodies to CD9, DPP4, and/or TMPRSS2. Following secondary antibody incubation and amplification of ligated oligonucleotide templates, punctate fluorescent DNAs were detected by confocal microscopy and counted using Imaris version 6.3.1 software.

**Fig 3:**
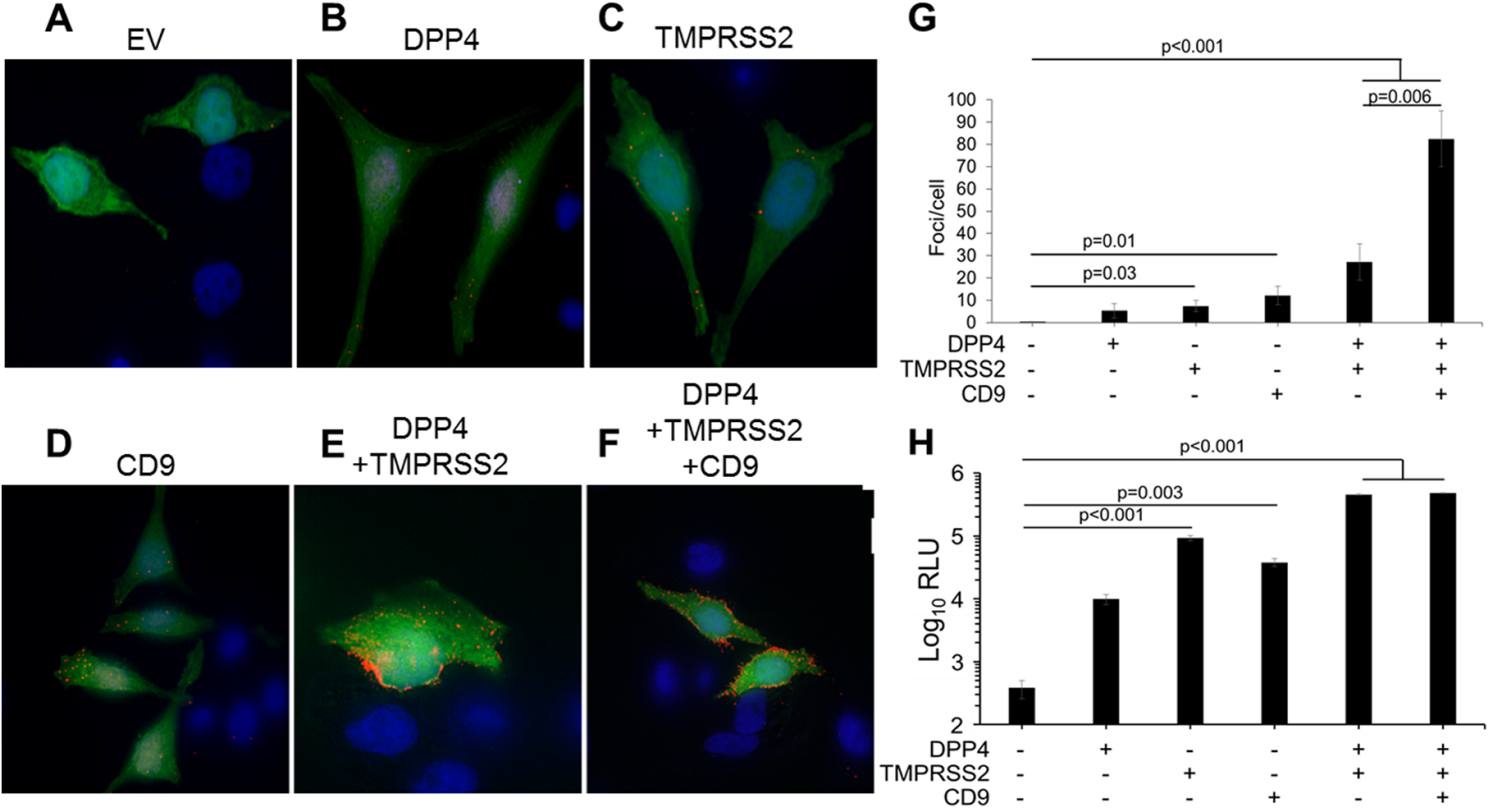
Proximity ligation assay of DPP4 and TMPRSS2 in CD9KO cells. (A-F) HeLa CD9KO cells were transfected with the indicated genes and a GFP reporter before being mounted on microscopy slides. Proximity ligation assay was performed using primary antibodies against hDPP4 and hTMPRSS2. Red foci indicate close proximity of the two proteins. (G) The average number of foci/cell in GFP^+^ cells in each group was quantified. (H) MERSpp transduction of HeLa cells overexpressing the indicated proteins.

Using hDPP4 and hTMPRSS2 antibodies, fluorescent foci were rarely observed on the HeLa-CD9KO cells (Fig 3A), and the cells were only modestly susceptible to MERSpp transduction (Fig 3H, leftmost bar). When CD9 was replenished in the CD9KO cells, foci were ~10-fold more abundant (Fig 3D), and these increased foci correlated with a greater cell susceptibility to MERSpp transduction (Fig 3H). These findings argue that CD9 sensitizes cells to MERS-CoV entry by bringing DPP4 and TMPRSS2 into proximity. We considered whether this role for CD9 applied only when DPP4 and TMPRSS2 levels were low, i.e., at endogenous HeLa-cell levels. Thus, hDPP4 and hTMPRSS2 were forcibly overexpressed; with overexpression, ~ 30 foci/cell were observed (Fig 3E), and this increased to ~ 80 foci/cell in the presence of CD9 (Fig 3F). MERSpp entry into cells correlated with the number of foci present, at least for values up to ~ 30 foci/cell (Fig 3H). Overall, these results indicated that CD9 connects DPP4 and TMPRSS2 entry factors, and is necessary for their proximity when they are sparse on cell surfaces. The CD9:DPP4:TMPRSS2 complexes then function as MERS-CoV entry portals. CD9 also helped to connect overexpressed DPP4 and TMPRSS2 together, but in this overexpression condition, CD9 did not increase MERSpp transduction, perhaps because other tetraspanins come in to bridge the abundant receptors and proteases. These results also revealed CD9-directed DPP4:TMPRSS2 complexes on intact cells in the absence of virus, suggesting that the CoVs infect through pre-existing complexes.

### MERSpps take a late endosomal entry route into CD9KO cells

**Fig 4:**
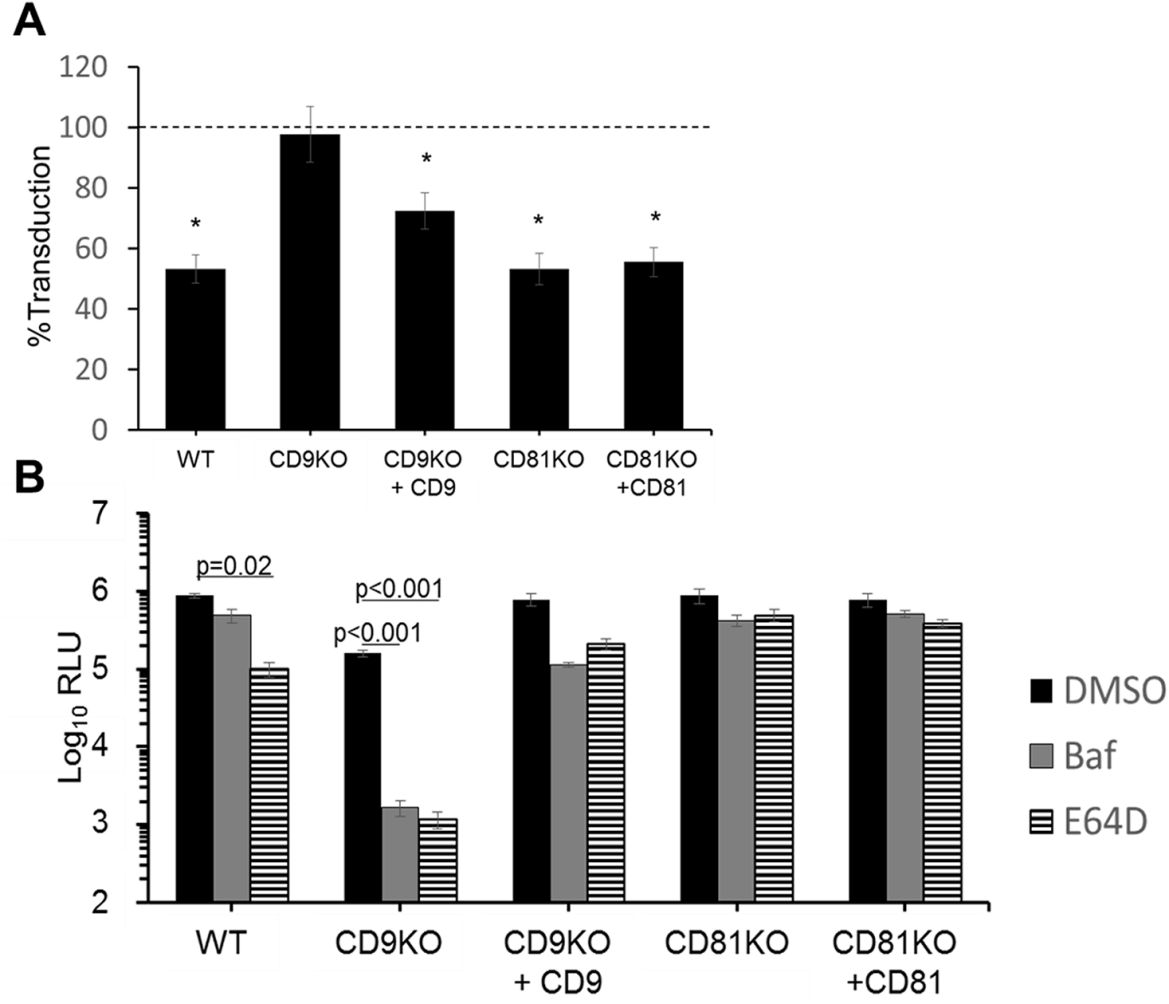
Protease sensitivity of MERS transduction in tetraspanin KO cells. (A) WT or KO cells were transfected with DPP4 and either an empty vector or the complementing tetraspanin as indicated. The cells were pretreated with camostat before transduction with MERSpps. MERSpp entry was measured by luciferase assays, and the percent transduction into camostat -treated cells was plotted relative to untreated cells (dotted line). * p<0.05. (B) WT or KO cell lines were transfected with DPP4 and either an empty vector or the complementing tetraspanin as indicated. The cells were pretreated with bafilomycin or E64D before transduction with MERSpp. MERSpp entry was measured by luciferase assay.

Because CD9 brought DPP4 in proximity with TTSPs, we hypothesized that CD9 facilitates TTSP-mediated early cell entry at or near plasma membranes, but does nothing to support the late, endosomal route that is enabled by cathepsin proteases. To test this, we inactivated cellular TTSPs using camostat [25] and found that camostat suppressed MERSpp transduction into WT cells by ~50%, but did not affect transduction into CD9KO cells (Fig 4A). CD9 complementation modestly restored MERSpp sensitivity to camostat. Furthermore, CD81 had no effect, as MERSpp entry into CD81KO and CD81-positive cells were equally suppressed by camostat (Fig 4A). These data were consistent with CD9 specifically enabling TTSP-directed, early virus entry.

Without CD9, the MERSpp entry route may be directed to a late, endosomal stage in which cathepsins provide fusion-activating triggers. To test this, we blocked late entry in WT and CD9KO with 100 μM bafilomycin A (Baf), an inhibitor of endosome acidification, or with 10 μM E64D, a cysteine protease inhibitor. In WT cells, Baf did not significantly decrease MERSpp entry, while E64D decreased entry ~4-fold (Fig 4B). However, in CD9KO cells, these inhibitors were far more antiviral, decreasing entry 20- and 100-fold, respectively. Complementing CD9 back into the CD9KO cells restored the WT phenotype in which the inhibitors were only weakly antiviral (Fig 4B). These differential effects of the inhibitors were not observed in CD81KO or CD81-overexpressing cells (Fig 4B). We conclude that CD9 is necessary for TTSP-mediated MERS early entry.

### Adenovirus vectors identify MERS-CoV entry factors in mice

**Fig 5:**
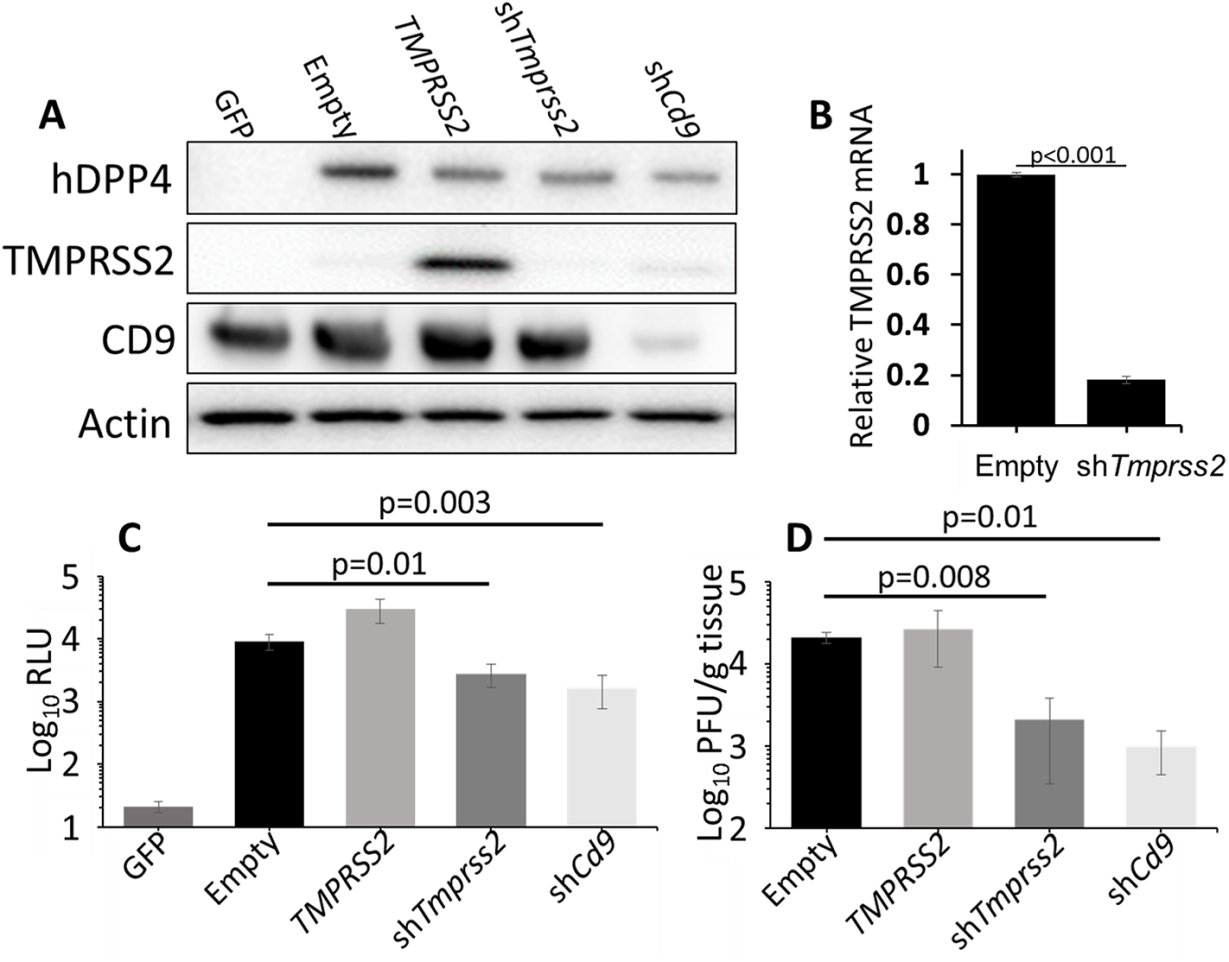
Analysis of Adenovirus knockdown of MERS entry factors. (A) LET-1 cells were transduced with an adenovirus carrying a GFP gene or adenoviruses carrying hDPP4 and either an empty vector, the TMPRSS2 gene, or a U6-driven shRNA against TMPRSS2 or CD9. After 3 days, cells were lysed and analyzed by western blot for the indicated proteins. (B) Quantitative rtPCR analysis of *Tmprss2* transcripts in cells transduced with rAd5-hDPP4-EV and rAd5-hDPP4-sh*Tmprss2*. (C) LET-1 cells were transduced with the indicated Ad5 vector before transducing with VSV-MERSpp. Transduction was measured by luciferase assay. (D) The indicated Ad5-DPP4 vectors were installed intranasally in C57/Bl6 mice. 5 days later, mice were infected with MERS-CoV. Lungs were isolated at 2 dpi and viral titers were measured by plaque assay.

We advanced to evaluating MERS-CoV entry factors *in vivo*. Of note, a previous study has demonstrated that camostat inhibits SARS-CoV spread in mouse lungs [36], suggesting that the virus exhibits dependence on serine proteases, probably TTSPs, for its entry *in vivo*. However, the importance of specific TTSPs, or for tetraspanins, is unknown for any *in vivo* CoV infection. Here we established infections in the mouse lung under conditions in which putative CoV entry factors were reduced. To do this, we developed dual-expressing recombinant adenovirus 5 (rAd5) vectors expressing both human DPP4, which sensitizes mouse cells to MERS-CoV infection [26, 37, 38], and shRNAs that knock down *Tmprss2* or *Cd9* mRNAs. In initial experiments, these rAd5 vectors were transduced into mouse Lung Epithelial Type 1 (LET-1) cells, a line derived from C57/Bl6 mouse alveolar type 1 cells [39]. After 3-days, the cells were analyzed for the presence of hDPP4, TMPRSS2, and CD9 by western blot (Fig 5A). Relative to the control rAd5-GFP transductions, all single and dual-expressing rAd5-hDPP4 transductants contained recognizable DPP4 and TMPRSS2, and those Ad5 vectors expressing shRNAs reduced the levels of endogenous CD9 proteins (Fig 5A). Due to endogenous TMPRSS2 protein levels being too low for detection on immunoblots, we used qRT-PCR to quantify TMPRSS2 transcripts. LET-1 cells transduced with rAd5-hDPP4-sh*Tmprss2* had only 25% of the transcripts of cells transduced with rAd5-hDPP4-empty vector (Fig 5B). This level of *Tmprss2* transcripts indicated an efficient knockdown of TMPRSS2 in the approximately 75% of cells that were successfully transduced. These results indicate that the different rAd5 vectors, transduced into cells derived from mouse alveolar epithelia, consistently express equivalent levels of hDPP4, while simultaneously increasing or decreasing TMPRSS2 or CD9.

To determine whether the rAd5-transduced LET-1 cells were susceptible to MERS-CoV S protein-directed virus entry, the cells were inoculated with recombinant VSVs encoding firefly luciferase [40] and pseudotyped with MERS-CoV S proteins. As expected, hDPP4 expression established susceptibility to VSV-MERSpp transduction (Fig 5C). TMPRSS2 co-expression from the Ad5 vectors increased susceptibility to MERSpps by ~ 4-fold, while sh*Tmprss2* and sh*Cd9* both restricted MERSpps by ~3 fold (Fig 5C). These results indicated that CD9 and TMPRSS2 act as entry factors in mouse lung-derived LET-1 cells, and suggested that the dual-expressing Ad5 vectors might be effective tools for identifying viral entry factors in the mouse lung.

To identify the role of CD9 and TMPRSS2 *in vivo*, the Ad5 vectors were instilled intranasally into mice which were, after 5 days, challenged with MERS-CoV. Lungs were harvested 2 days post-infection (d.p.i.) and MERS-CoV titers were measured as PFU/gram of tissue. Relative to MERS-CoV titers in rAd5-h*DPP4* transduced animals, the MERS-CoV titers in rAd5-h*DPP4*-sh*Cd9* transduced animals were ~20-fold lower (Fig 5D). Furthermore, the MERS-CoV titers in rAd5-h*DPP4*-sh*Tmprss2* transduced mice were reduced by ~10-fold. Interestingly, overexpression of TMPRSS2 by the rAd5-h*DPP4*-*TMPRSS2* vector had no effect on MERS-CoV titers in the lungs, presumably because the lung environment has sufficient endogenous murine TMPRSS2 to facilitate efficient MERS-CoV infection. These data indicate that CD9 and TMPRSS2 act as MERS-CoV susceptibility factors in the lung parenchyma and that their role in entry is slightly more pronounced *in vivo* than in *in vitro* LET-1 mouse alveolar cell cultures. Indeed, these data show that CD9 and TMPRSS2 are responsible for ~90% of MERS-CoV titers *in vivo*.

### Virulent MERS viruses utilize CD9-dependent early entry

**Fig 6:**
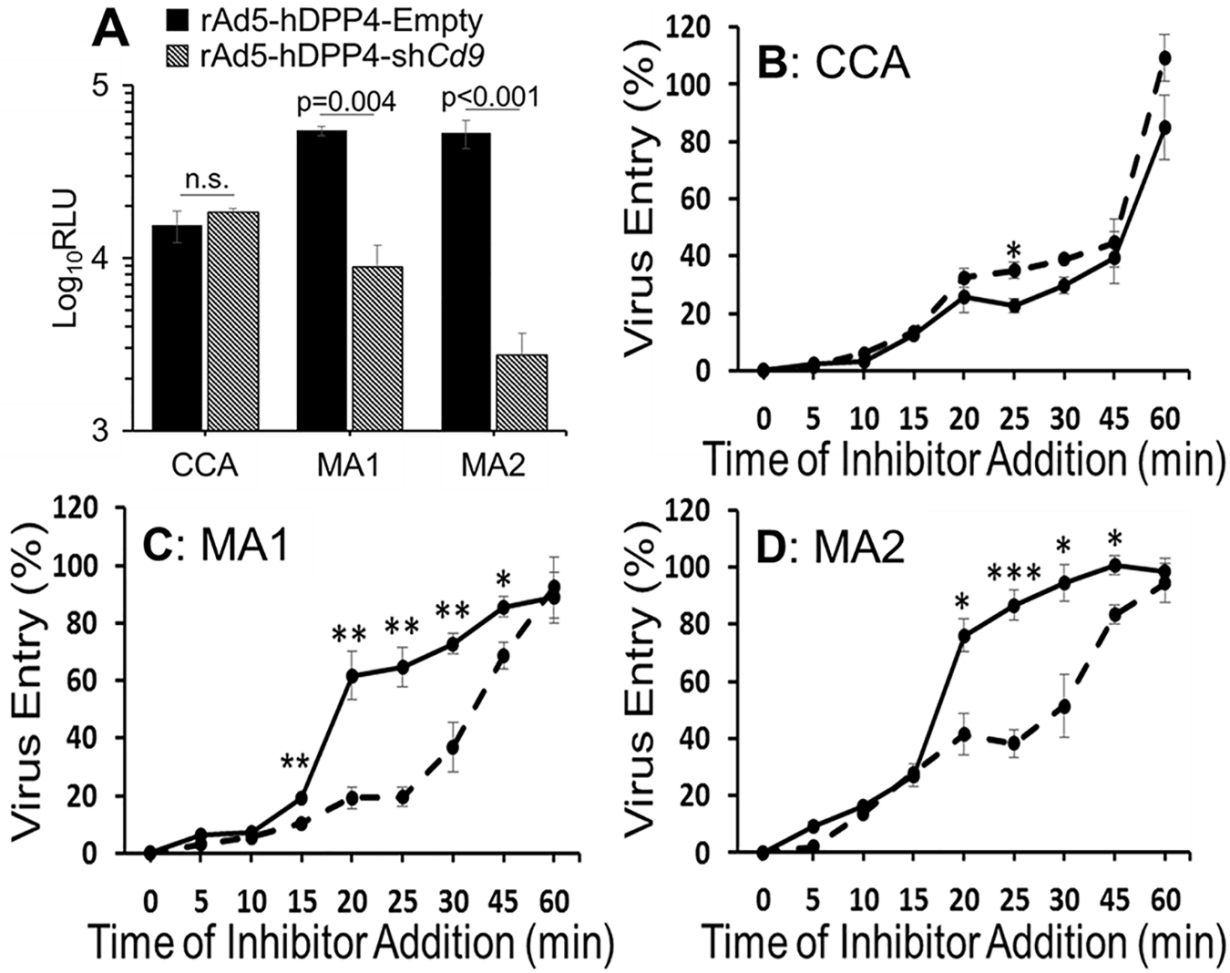
CD9 utilization of high virulence MERS viruses. (A)LET-1 cells were transduced with rAd5-hDPP4-Empty (black bars) or rAd5-hDPP4-sh*Cd9* (dashed bars) before transduction with VSV-pps carrying the indicated MERS S proteins. The entry kinetics of MERSpps carrying cell-culture adapted (B), MA1 (C), or MA2 (D) S proteins was measured in LET-1 cells previously transduced with rAd5-hDPP4-Empty (solid lines) or rAd5-hDPP4-shCd9 (dashed lines). Virus entry was calculated relative to a non-inhibitor treated condition. *p<0.05, **p<0.01, ***p<0.001

MERS-CoV, a camel and human virus [41, 42], has recently been adapted for robust growth and virulence in hDPP4^+^ mouse lungs [43, 44]. This adaptation process was initiated by intranasally infecting mice with avirulent, Vero Cell Culture-Adapted (CCA) MERS-CoVs and then serially passaging viruses through hDPP4^+^ mouse lungs. Relative to CCA MERS-CoVs, the Mouse-Adapted (MA) viruses have distinct S protein changes [44](S2 Table). We considered whether these MA changes fixed into S proteins adapt viruses to utilize CD9-facilitated early entry. To address this question, we produced VSV-based MERSpps, pseudotyped with the CCA or MA S proteins. These CCA and MA MERSpps were transduced into CD9-replete or CD9-knocked down (CD9KD) LET-1 cells. The CD9-replete and CD9KD cells were equally susceptible to CCA S-mediated pp entry. However, the same CD9KD cells had 90% and >95% reduced susceptibility to MA1 and MA2 S-driven pp entry, respectively (Fig 6A). Thus, it appears that *in vivo* passage in mouse lungs adapts MERS-CoVs to a CD9-facilitated cell entry pathway.

The MA viruses’ utilization of CD9 for entry correlated with their relatively rapid entry kinetics [44]. Furthermore, CD9-facilitated entry correlates with TTSP utilization (Fig 4), and TTSP utilization correlates with rapid CoV entry into cells [45]. Therefore, we hypothesized that CD9 is a determining factor in CoV-cell entry kinetics. To test this, CCA and MA MERSpps were transduced into CD9-replete or CD9KD LET-1 cells. To measure pp entry kinetics, the transduction process was abruptly halted at defined time points with a nontoxic protease inhibitor cocktail that prevents S-directed fusion, but has no effect on transduced reporter gene expression [45]. This strategy allows Fluc reporter accumulations to indicate the extents of virus entry taking place within timed intervals.

We found that CD9 had no influence on the rate of CCA S-directed virus entry. In both CD9-replete and CD9-KD cells, half-maximal entry was complete within 45 min (Fig 6B). A 30–45 min half time for virus entry is found for several viruses requiring endocytosis prior to genome delivery [46]. However, CD9 strongly influenced MA S-mediated virus entry. The MA1 and MA2 pps reached 50% entry in CD9-replete cells in 20 and 19 minutes, but were delayed to 34 and 30 minutes, respectively, in CD9KD cells (Fig 6C and 6D). These data were corroborated with the tetraspanin KO cell lines (S4 Fig). Thus, we conclude that CD9 utilization and rapid cell entry correlate with mouse adaptation and MERS virulence in mouse lung infections.

## Discussion

In this study, we demonstrated that the MERS coronavirus enters cells using an entry complex that includes a receptor, a protease and a tetraspanin. The tetraspanin operates by bringing the receptor and protease(s) into proximity, such that viral spikes, once attached to receptors, are quickly and efficiently cleaved into fusion-activated forms. These ternary complexes pre-exist on virus-target cells and can theoretically have highly variable subunit composition. Presently, there are three known human CoV receptors, 17 human transmembrane serine proteases, and 34 human tetraspanins, and therefore there are thousands of potential combinations of receptor, protease and tetraspanin that might provide a coronavirus entry platform.

For the MERS coronavirus, a particularly effective complex includes the hDPP4 receptor, the protease TMPRSS2, and the tetraspanin CD9. This was discovered, in large part, through creative use of recombinant adenoviruses (rAds). The approaches used here extended from the finding that a transducing rAd5 expressing the MERS-CoV receptor hDPP4 sensitized laboratory mice to MERS-CoV infection [26]. By incorporating RNA silencing genes into rAd5-hDPP4, we were able to simultaneously establish MERS-CoV susceptibility, through hDPP4 expression, and potentially restrict MERS-CoV, through shRNA-mediated suppression of candidate proviral factors. Thus, the dual-expressing rAd5 vectors revealed CD9 and TMPRSS2 as relevant proviral factors, operating to support the primary hDPP4 susceptibility factor. It is notable that dual-expressing adenovirus vectors can potentially be utilized to identify any MERS-CoV host factor. Indeed, they can be utilized to identify host factors in any virus-host system that requires an exogenously supplied susceptibility determinant. Furthermore, the adenovirus transduction process bypasses the need to establish partially humanized mice for studies of human coronavirus infections, and actually has distinct advantages over transgenic animals, in that shRNAs reduce pro- or anti-viral factors solely in coronavirus-infectable cells, making for reliable measurements of changes in virus susceptibility. We expect that dual-expressing rAd5 vectors will be excellent general tools to rapidly identify *in vivo* pro- and anti-viral host factors.

The hDPP4:CD9:TMPRSS2 complexes promoted an “early” MERS-CoV entry. CoV S proteins require simultaneous receptor engagement and proteolysis before catalyzing virus-cell membrane fusion [3, 47, 48], a process demanding that TMPRSS2 be closely juxtaposed to hDPP4. We suggest that CD9 tetraspanins position TMPRSS2 next to the receptor-bound S proteins, perhaps in association with cholesterol, a lipid having profound effects on both tetraspanin structural interactions [49] and CoV entry [50–54]. Precisely how TMPRSS2 abuts against hDPP4 to access S proteins is not clear, although the structures of three CoV S proteins [55, 56] and hDPP4 [57] indicate that the proteolytic cleavage sites would be displayed at the outer edges of each S trimer. Furthermore, it is likely that proteolytic cleavage of several adjacent S proteins is needed to activate membrane fusion, as cooperative “pulling” by several viral fusion proteins is frequently required for virus entry processes [12, 13, 58]. Therefore, the tetraspanin-enriched environment, in which DPP4 and TMPRSS2 are collected together, likely permits rapid and simultaneous cleavage of multiple, closely-spaced virion S proteins, generating clusters of activated S proteins that drive the membrane fusion process.

Without CD9, the hDPP4 and TMPRSS2 are not held closely together (Fig 3). In this condition, MERS-CoV still infects hDPP4-positive target cells (Fig 3), but it takes a slower “late” endosomal route, which we and others find to be around 90% less efficient than early entry (Figs 1, 5 and 6) [25]. In the late entry route, virus-associated S proteins are first endocytosed and then cleavage-activated by furin proprotein convertases [59, 60], cathepsin L [8, 25, 61, 62], and/or cathepsin B [63]. However, the protease-enriched endo-lysosomal environment [64] can also generate inactivating CoV S protein cleavages, as evidenced by C-terminal S protein fragments, 40 kDa and smaller, that must be inactivated fusion domain fragments [48, 65]. Therefore, in the late entry route, there may be a short time span between a cathepsin-activated fusogenic state and a permanently inactivated, excessively proteolyzed state, accounting for inefficient entry. Inefficient late entry may also be explained by differences in lysosomal and plasma membranes, which have unique lipid profiles [66] and therefore may be differentially susceptible to S -catalyzed fusion. Finally, late entry is restricted by interferon-induced gene products, notably interferon-induced transmembrane (IFITM) proteins [67, 68], but early TTSP-facilitated entry is not [8]. All of these virus-restricting conditions may combine *in vivo* to make CD9-facilitated “early” cell entry the predominant route for MERS-CoV infections.

That the TTSP-facilitated entry route is predominant *in vivo* is supported by the recent finding that serine protease inhibitors reduce SARS-CoV infection in mouse lungs [36]. Additionally, clinical HCoV-229E isolates use a rapid TTSP-facilitated entry route, unlike lab-adapted HCoV-229Es [45]. More recently, similar patterns were observed for MERS-CoVs. Mouse lung-adapted MERS-CoVs take a rapid TMPRSS2-mediated cell entry, while cell culture-adapted (CCA) MERS-CoVs are avirulent and enter cells through the slower and less efficient endocytic route [44]. Here we demonstrated that the virulent MA MERS-CoV S proteins utilized CD9 during cell entry, while avirulent CCA viruses did not. This new finding suggests that CoV receptors and proteases alone are not the selective agents in CoV adaptation. Rather, the CoVs adapt to the ternary receptor-tetraspanin-protease complexes. In the case of MERS-CoV, the key adaptive S mutation facilitating the usage of the ternary complexes was at position 1015 (S2 Table). In the MERS-CoV S protein cryo-EM structure [55, 56, 69], this residue 1015 is part of a peptide that connects two of the helices comprising the fusion domain. The change from N1015 to T may ease restrictions to conformational change in the S trimer, thereby exposing cleavage sites to the nearby CD9-associated transmembrane protease, with cleaved spikes then converting to fusion-active forms.

Finally, these findings may shed light on general roles for tetraspanins in virology. Four CoV receptors (DPP4, APN, ACE2, and CEACAM) were found in tetraspanin-rich membrane fractions (Fig 4), and our previous report indicated that tetraspanin antibodies block several CoV infections by interfering with receptor-associated CoV access to surface proteases [14]. Even antibodies binding to CD81 suppressed MERS S-mediated entry [14], indicating that several tetraspanins, including those that are not required *per se* for clustering hDPP4 and TMPRSS2, organize into cell-surface “webs” [15] and enclose the CoV entry factors. Here, there may be parallels with several tetraspanin-facilitated viruses, including influenza A (IAV) [28] and canine distemper (CDV) [70]; the retroviruses HIV [71, 72], feline immunodeficiency virus (FIV) [73], and human T-lymphocytic virus 1 (HTLV-1) [74]; herpes simplex virus 1 (HSV-1) [75]; hepatitis C virus [76]; and several human papillomaviruses (HPVs) [77]. For these viruses, tetraspanins facilitate viral entry (CoVs, IAVs, HCV, HPVs) syncytia formation (CDV, HIV, FIV, HTLV-1), or promote viral exit (IAVs, HSV-1 and HIV), by unclear mechanisms. Conceivably, a common mechanism may involve tetraspanin-mediated clustering of host factors. For example, tetraspanin CD81 is both an HCV receptor [78] and a linker of the HCV co-receptors scavenger receptor class B I (SR-BI) [76] and claudin-1 [79], whose complexing promotes viral endocytosis (reviewed in [80]). Another example is with the tetraspanins CD151 and CD63, which do not directly interact with HPVs, but rather hold several co-receptors together to permit HPV binding and endocytosis (reviewed in [81]. Therefore, many of the proviral activities ascribed to tetraspanins may relate to their ability to cluster transmembrane proteins, as we have found for the pro-MERS-CoV activity of CD9. Given that several viruses depend on tetraspanin webs, it may be useful to consider ways to target entry-blocking drugs to these locations and thereby increase their antiviral efficacy.

## Methods

### Mice, virus, and cells

C57BL/6 mice were purchased from the National Cancer Institute and housed in the animal care facility at the University of Iowa. The MERS-CoV (EMC2012 strain) was provided by Drs. Bart Haagmans and Ron Fouchier (Erasmus Medical Center). HEK293T and HeLa cells were obtained from Dr. Edward Campbell (Loyola University Chicago) and maintained in Dulbecco's Modified Eagle Media (DMEM) supplemented with 10% fetal bovine serum (FBS, Atlanta Biologicals), 10 mM HEPES, 100 mM sodium pyruvate, 0.1 mM non-essential amino acids, 100 U/ml penicillin G, and 100 μg/ml streptomycin. LET-1 cells were obtained from BEI Resources and were maintained in DMEM supplemented with 10% FBS, 100 U/ml penicillin G, and 100 μg/ml streptomycin. Cells were maintained in a humidified environment at 37°C and 5% CO_2_. HAE cultures were isolated and maintained as described previously [48].

### Ethics statement

This study was carried out in strict accordance with the recommendations in the Guide for the Care and Use of Laboratory Animals of the National Institutes of Health. Animal experiments were approved by the Institutional Animal Care and Use Committee at the University of Iowa (Protocol #4041009).

### Plasmids

Codon-optimized MERS-CoV S containing a C9 tag was purchased from Genscript and subsequently cloned into pcDNA3.1+ between the EcoRI and NotI restriction sites. pcDNA3.1-229E-Spike-C9 and pcDNA3.1-hAPN plasmids were provided by Dr. Fang Li, (University of Minnesota). pcDNA3.1-SARS-Spike-C9 and pcDNA3.1-ACE2-C9 plasmids were provided by Dr. Michael Farzan (Scripps Research Institute). C-terminal FLAG-tagged human DPP4 plasmid pCMV6-Entry-hDPP4 (NCBI Reference Sequence NM_001935) was purchased from OriGene. pCAGGS-TMPRSS2-FLAG was previously constructed [82]. The pNL4.3-HIVluc plasmid was provided by the NIH AIDS Research and Reference library. pCMVSport6-hCD9 was purchased from Open Biosystems. pSpCas9-BB-2A-puro was a gift from Feng Zhang (Addgene plasmid # 52961). psPAX2 was a provided by Dr. Ed Campbell (Loyola University Chicago). For transfections, plasmid DNAs were incubated with polyethylenimine (PEI, Polysciences Inc., Warrington, PA), at 1:3 DNA:PEI mass ratio, in Opti-MEM (Life Technologies, Carlsbad, CA) for 15 min at room temperature (RT), then added dropwise to adherent cells (2 μg DNA per 10^6^ cells).

### Antibodies

Monoclonal mouse antibodies against CD9 (clone M-L13), CD63 (clone H5C6), and CD81 (clone JS-81) were obtained from BD Pharmingen. Rabbit anti-FLAG was obtained from Sigma Aldrich. Mouse anti-rhodopsin antibodies were obtained from Millipore. Rabbit anti-CD13 (APN) antibodies were obtained from Abcam. Mouse anti-CD26 (clone M-A261) was obtained from BD Biosciences. Rabbit anti-TMPRSS2 (clone EPR3681) was obtained from Abcam. Secondary antibodies were purchased from Invitrogen and include goat-anti-rabbit-AlexaFluor 488, goat-anti-mouse-AlexaFluor 488, and goat-anti-mouse-AlexaFluor 568. Donkey-anti-goat, goat-anti-mouse, and goat anti-rabbit HRP conjugated antibodies were purchased from Thermo Scientific.

### Recombinant adenovirus production

Recombinant adenovirus vectors were produced as previously described by the University of Iowa Gene Transfer Vector Core [83]. To generate TMPRSS2-expressing adenoviruses, hTMPRSS2 containing a C-terminal FLAG tag was cloned into the pAd5CMV shuttle vector between XhoI and EcoRI restriction sites. To generate shRNA—expressing adenoviruses, gene blocks containing an shRNA targeting either the coding region of CD9 (target sequence: CCGATTCGACTCTCAGACCAA) or the 3' UTR of TMPRSS2 (target sequence: ACACTAGAGTGGATGAATGTCTGGA), flanked by the U6 promoter and RNApolIII termination sequence, were purchased from GenScript. These gene blocks were subcloned into the pacAd5k-NpA E1 shuttle vector between the KpnI and EcoRI restriction sites. Shuttle vectors were linearized and transfected into HEK 293 cells along with a linearized Ad5 backbone containing an RSV promoter -driven h*DPP4* in the E3 region. Homologous recombination in HEK 293 cells yielded recombinant adenovirus encoding both the shRNA and hDPP4. Titers of purified recombinant adenoviruses ranged from 10^10^−10^11^ pfu/ml.

### Transduction and infection of mice

Isoflurane-anesthetized mice were transduced intranasally with 2.5 x 10^8^ pfu of the indicated Ad5 virus in 75 μl of DMEM. 5 days posttransduction, mice were infected intranasally with 10^5^ pfu of MERS-CoV in a total volume of 50 μl DMEM. At 2 d.p.i., mice were euthanized by isoflurate inhalation followed by cervical dislocation. Lungs were removed into PBS and manually homogenized. Virus was plaqued on Vero81 cells. Cells were fixed with 10% formaldehyde and stained with crystal violet 3 d.p.i. All work was performed in the University of Iowa Biosafety Level 3 (BSL3) Laboratory.

### HIV and VSV-based pseudoviruses

HIV pseudoviruses were produced as previously described [84]. Briefly, 293T cells were co-transfected with pNL4.3-HIV-luc and pcDNAs encoding appropriate glycoproteins. After two days, supernatants were collected, centrifuged at 10,000 x g at 4°C for 10 minutes to remove debris, and stored in aliquots at -80°C. VSV pseudoviruses were produced by the methods of Whitt, 2010 [40]. Briefly, 293T cells were transfected with plasmids encoding viral glycoproteins. Two days later, cells were inoculated for 2h with VSVΔG-luciferase [40], rinsed extensively and incubated for one day. Supernatants were collected, centrifuged at 10,000 x g at 4°C for 10 minutes to remove cellular debris, and stored in aliquots at -80°C.

Pseudovirus transductions were carried out by incubating target cells with pseudoviruses for 1h at 37°C. Following initial incubation, unadsorbed viruses were removed by washing thrice with PBS. Complete media was placed on the cells and incubated for 18h for VSV or 48h for HIV at 37°C. At the end of transduction periods, cells were dissolved into cell culture lysis buffer (25 mM Tris-phosphate [pH 7.8], 2 mM DTT, 2 mM 1,2-diaminocyclohexane-N,N,N ′,N ′-tetraacetic acid, 10% glycerol, 1% Triton X-100) and luciferase levels were measured by addition of firefly luciferase substrate (1 mM D-luciferin, 3 mM ATP, 15 mM MgSO4·H2O, 30 mM HEPES [pH 7.8]) using a Veritas microplate luminometer (Turner BioSystems, Sunnyvale, CA).

### Production of knockout cell lines

pSpCas9-BB-2A-puro was digested with Esp3I (Fermentas) for 4h at 37°C. The digested plasmid was purified and ligated with annealed guide DNAs specific for CD9 or CD81. Tetraspanin-specific pSpCas9-BB-2A-puro plasmids were transfected into 293T cells. After 72h, cells were selected with 4 μg/ml puromycin for 96h. Selected cells were serially-diluted to isolate clonal populations and clones were selected by western blot.

### Tetraspanin enriched membrane isolation

Adherent 293T cells (~10^5^ / cm^2^) were rinsed with ice-cold PBS, incubated for 30 min at 4°C with PBS-1 mg/ml EZ-Link Sulfo-NHS-LC-Biotin (Pierce), then for 20 min at 4°C with PBS-100 mM glycine. Cells were rinsed with PBS, then incubated for 20 min at 4°C in MES buffer (25 mM MES [pH 6.0], 125 mM NaCl, 1 mM CaCl_2_, 1 mM MgCl_2_) containing 1% 3-[(3-Cholamidopropyl)dimethylammonio]-1-propanesulfonate (CHAPS) detergent (Calbiochem Cat # 220201) or 1% Triton X-100 detergent (Sigma). Cell lysates (10^7^/ml) were removed from plates and emulsified by 20 cycles of extrusion through 27G needles. Nuclei were removed by centrifugation, lysates mixed with equal volumes of 80% w/v sucrose in MES buffer, placed into Beckman SW60 tubes, and overlaid with 3 ml of 30% w/v sucrose, then with 0.5-ml of 5% w/v sucrose, both in MES buffer. Samples were centrifuged with a Beckman SW60 rotor at 370 K x g for 18 h at 4°C. Fractions were collected from air-gradient interfaces. Biotinylated proteins in gradient fractions were bound to streptavidin agarose beads (Pierce). Non-reducing western-blotting procedures were used to identify the distributions of proteins in gradient fractions, as described previously [38].

### Proximity ligation assay

HeLa cells were transfected with indicated plasmid DNAs and a GFP reporter, incubated for two days, and then lifted from tissue culture plates using 0.05% trypsin. Cells were transferred to microscope coverslips coated with fibronectin. Cells were allowed to adhere for 24h. Cells were then fixed for 30 minute at 37°C with 3.7% paraformaldehyde in 0.1 M piperazine-N,N′-bis(2-ethanesulfonic acid) buffer (pH 6.8). Coverslips were washed with PBS and PLA was performed using DuoLink Proximity Ligation Assay (Sigma-Aldrich) using primary antibodies against TMPRSS2 and CD26. Images were captured using a DeltaVision microscope (Applied Precision) equipped with a digital camera (CoolSNAP HQ; Photometrics), using a 1.4-numerical aperture 60X objective lens. Images were deconvoluted with SoftWoRx deconvolution software (Applied Precision). PLA foci were detected and quantified using Imaris version 6.3.1 (Bitplane Scientific Solutions).

### Entry kinetics assay

293T cells were transfected with DPP4 and either an empty vector or complementing tetraspanin. 24h after transfection, cells were plated in a 96-well plate. MERSpps were added to cells at 4°C for 1 hour to allow viral binding. Media was removed and replaced with 37°C media and the plates were moved to an incubator. At sequential time points following the shift to 37°C, a protease inhibitor cocktail was added to cells such that the final concentration was 100 μM camostat, 10 μM proprotein convertase inhibitor, and 10 μM E64d. These drugs were left on cells overnight before cells were lysed and luciferase was measured as described above. Luciferase levels were compared to that of cells treated only with DMSO control.

### Protease inhibitor assays

293T cells were transfected with DPP4 and an empty vector or the complementing tetraspanin. Cells were pre-treated for 1h with 100 μM camostat, 100 μM bafilomycin, or 10 μM E64D before transduction with MERSpps in the presence of the inhibitors. After 2h, cells were washed to remove drugs and unadsorbed virus. Luciferase assays were performed as described above.

### Quantitative reverse transcriptase-PCR

Cellular RNA was isolated using the RNeasy Mini Kit (Qiagen) and 100 or 500 ng was reverse transcribed using an iScript cDNA synthesis kit (Bio-Rad). Quantitative PCR was performed using Power SYBR Green (Thermo Fisher) and primers specific to human CD9, DPP4, TMPRSS2, or HPRT.

### Statistical analysis

Statistical comparisons were made by two-tailed Student’s t-test. Error bars in the figures indicate the standard error of the data. Non-linear regression analysis was used to fit a curve to the entry kinetics data and obtain the time of 50% infection. This analysis was performed using Minitab 17 software.

## Supporting information

S1 Fig CoV Transduction of CD81KO 293T cells. 293T WT or CD81KO cells were transfected with appropriate receptors with or without CD81. These cells were transduced with HIV pseudoviruses carrying the S proteins of MERS (A), 229E (B), SARS (C), or MHV(D). Pseudovirus transduction was measured using luciferase assay.
(TIF)

S2 Fig Effects of CD9 and TMPRSS2 expression on MERSpp entry in cells with endogenous DPP4. 293-WT (black) or 293-CD9KO (gray) cells were transfected with an empty vector (EV), CD9 (A), or TMPRSS2 (B) before transduction with MERSpp. MERSpp entry was measured by luciferase assay.
(TIF)

S3 Fig Association of tetraspanins with CHAPS-resistant membranes. 293T WT, CD9KO, and CD81KO cells were analyzed for tetraspanin distribution following differential centrifugation of CHAPS lysates. The CD9KO and CD81KO cells were complemented with the appropriate tetraspanins by transfection.
(TIF)

S4 Fig Entry kinetics of MERS-EMCpp in tetraspanin KO cells. The entry kinetics of MERSpps were measured in 293T WT, CD9KO (A), and CD81KO (B) cells. Cells were bound with MERSpps and incubated with entry inhibiting protease cocktail at the indicated time point. Luciferase levels were measured and plotted relative to untreated control cells. Entry kinetics into KO cells complemented with the appropriate tetraspanins are indicated by dotted lines. (C) The entry kinetics of MERSpps into KO cells overexpressing TMPRSS2. *p<0.01 compared to WT cells.
(TIF)

S1 Table Relative expression of CD9, DPP4, TMPRSS2 and HPRT in HeLa and human airway epithelia cells. (TIF)

S2 Table Amino acid substitutions in MERS mutants. (TIF)
