# Supplementary figures and images for: The tetraspanin CD9 facilitates MERS-coronavirus entry by scaffolding host cell receptors and proteases
Source: PLoS Pathog. 2017 Jul 31;13(7):e1006546. doi: 10.1371/journal.ppat.1006546 (PMC5552337; doi:10.1371/journal.ppat.1006546)

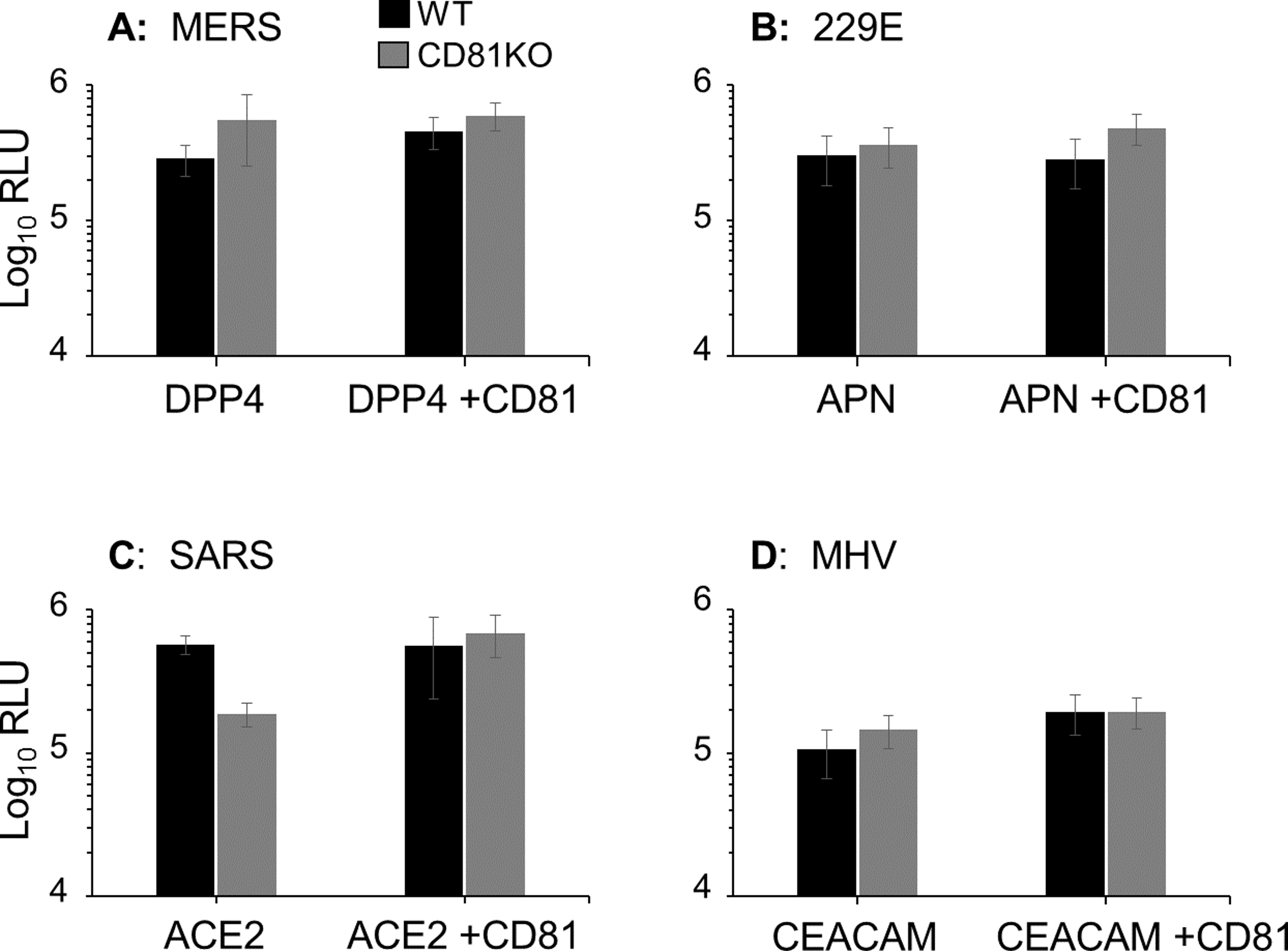

Supplement: S1 Fig — 293T WT or CD81KO cells were transfected with appropriate receptors with or without CD81. These cells were transduced with HIV pseudoviruses carrying the S proteins of MERS (A), 229E (B), SARS (C), or MHV(D). Pseudovirus transduction was measured using luciferase assay. (TIF) [file ppat.1006546.s001.tif]

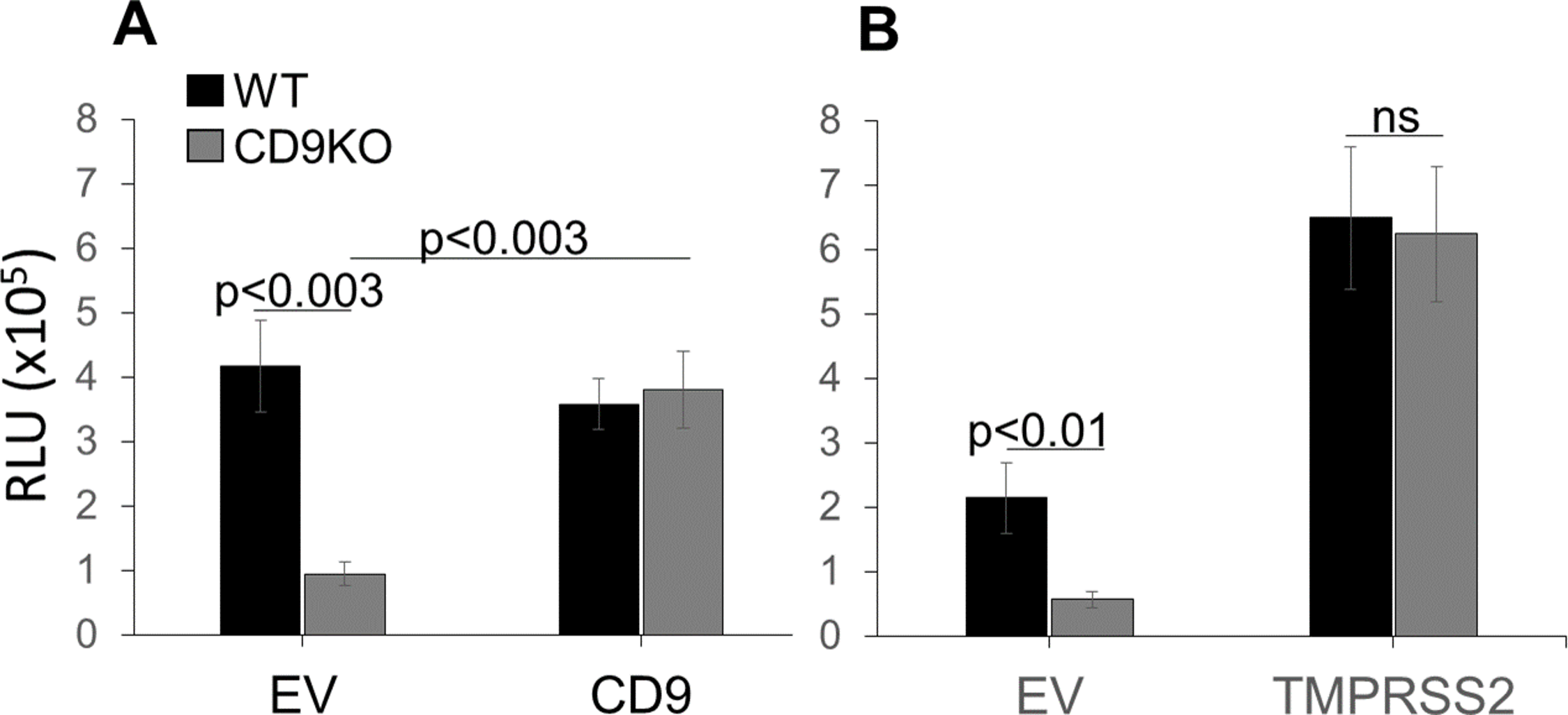

Supplement: S2 Fig — 293-WT (black) or 293-CD9KO (gray) cells were transfected with an empty vector (EV), CD9 (A), or TMPRSS2 (B) before transduction with MERSpp. MERSpp entry was measured by luciferase assay. (TIF) [file ppat.1006546.s002.tif]

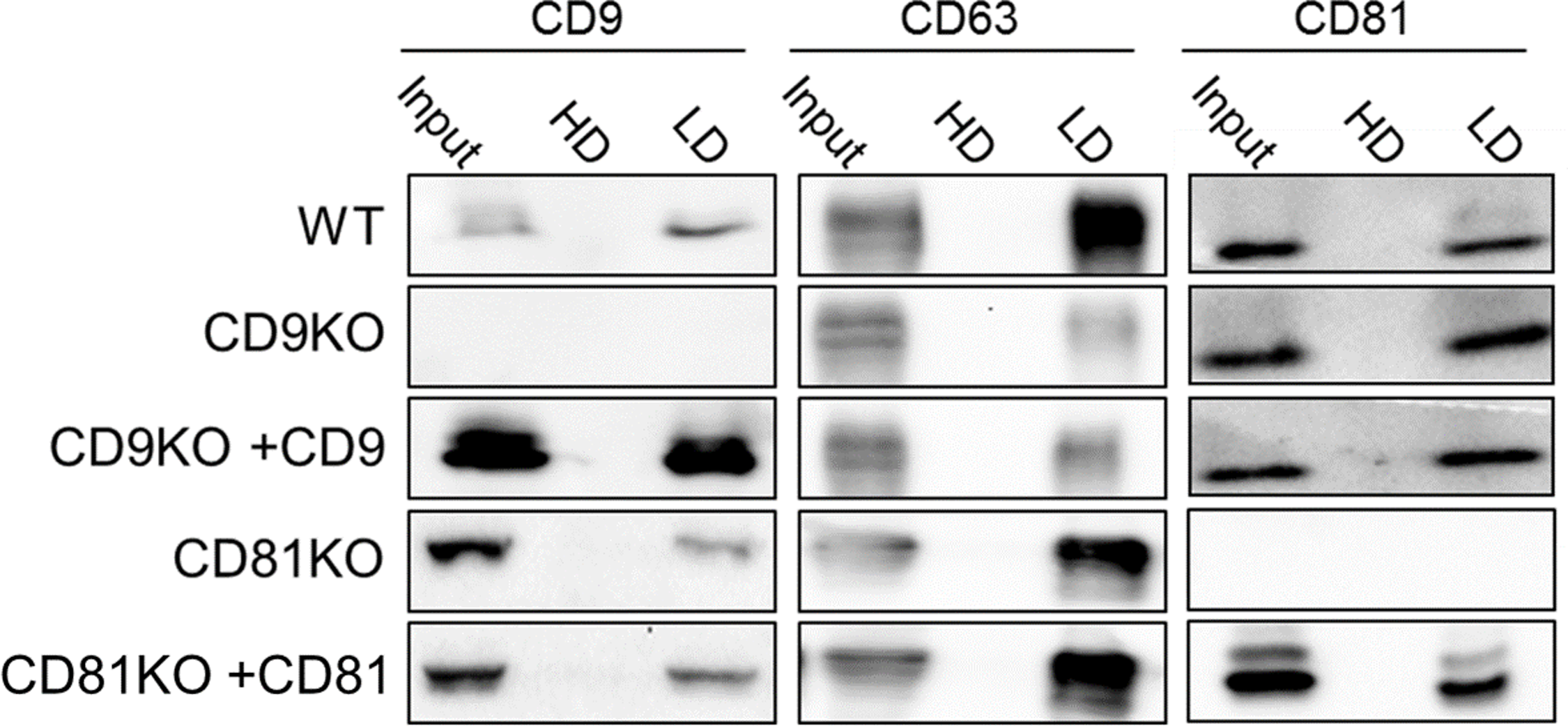

Supplement: S3 Fig — 293T WT, CD9KO, and CD81KO cells were analyzed for tetraspanin distribution following differential centrifugation of CHAPS lysates. The CD9KO and CD81KO cells were complemented with the appropriate tetraspanins by transfection. (TIF) [file ppat.1006546.s003.tif]

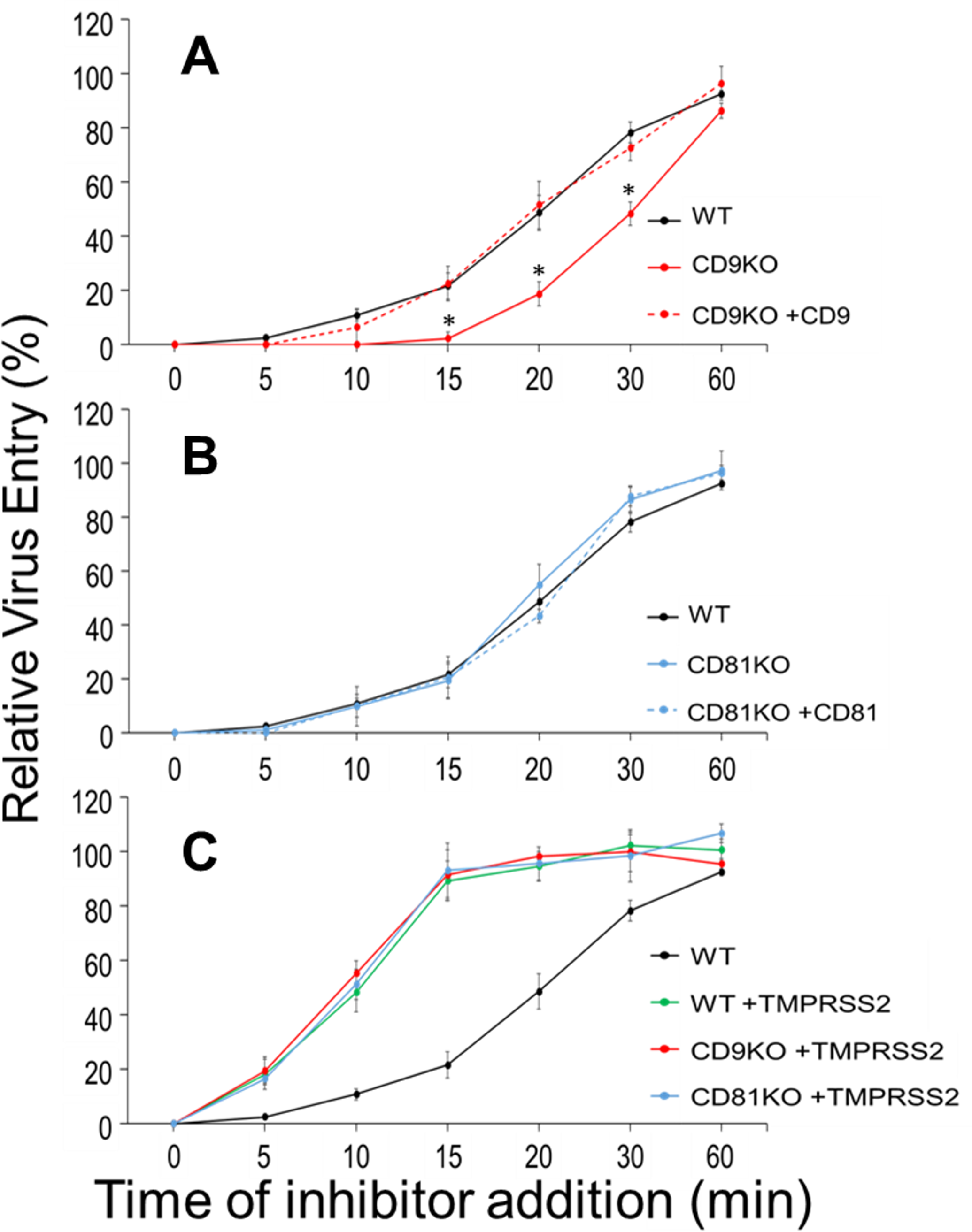

Supplement: S4 Fig — The entry kinetics of MERSpps were measured in 293T WT, CD9KO (A), and CD81KO (B) cells. Cells were bound with MERSpps and incubated with entry inhibiting protease cocktail at the indicated time point. Luciferase levels were measured and plotted relative to untreated control cells. Entry kinetics into KO cells complemented with the appropriate tetraspanins are indicated by dotted lines. (C) The entry kinetics of MERSpps into KO cells overexpressing TMPRSS2. *p<0.01 compared to WT cells. (TIF) [file ppat.1006546.s004.tif]

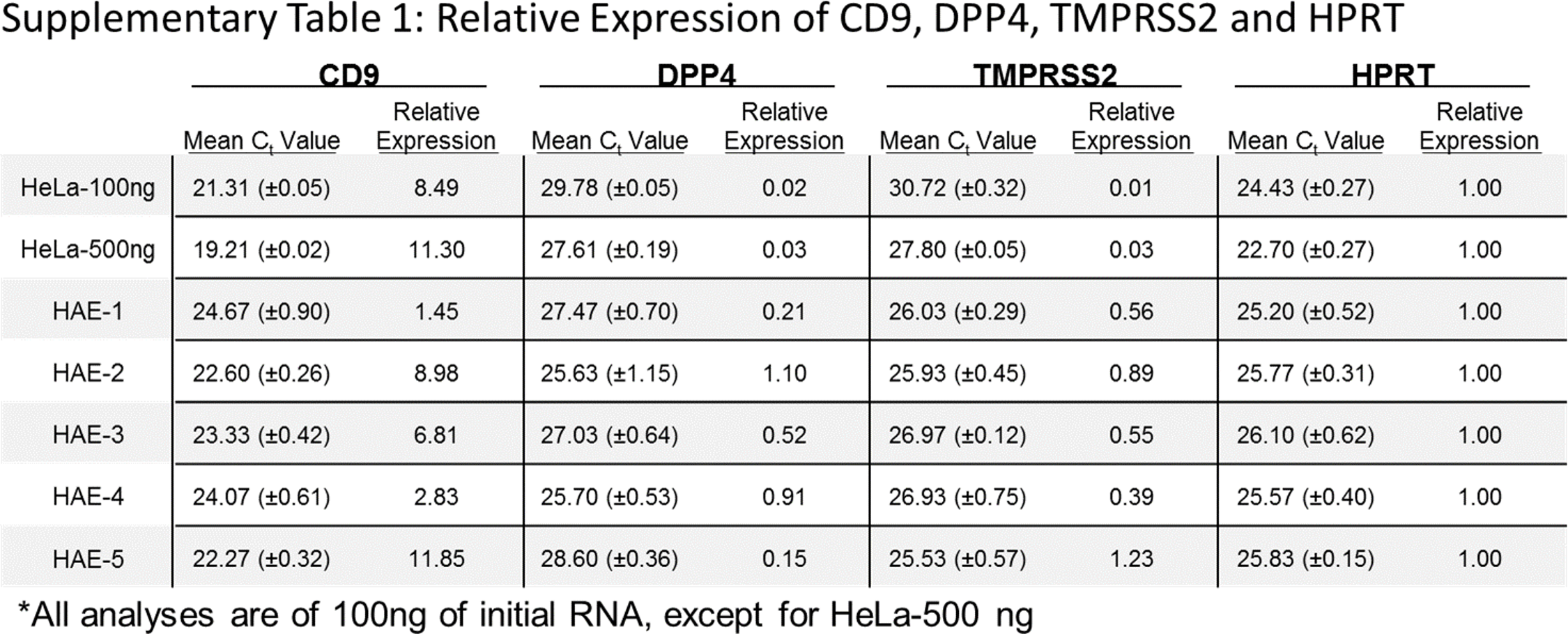

Supplement: S1 Table — (TIF) [file ppat.1006546.s005.tif]

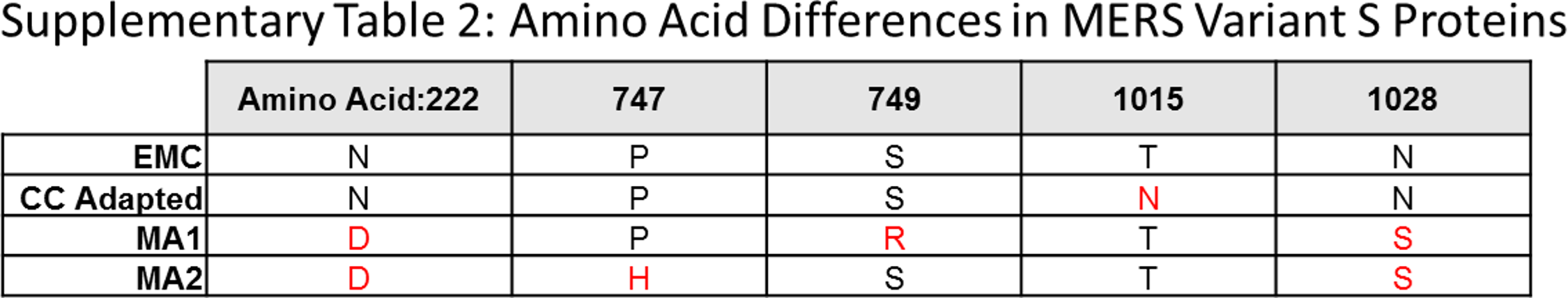

Supplement: S2 Table — (TIF) [file ppat.1006546.s006.tif]
